# Quarterly fluctuations in external and internal loads among professional basketball players

**DOI:** 10.3389/fphys.2024.1419097

**Published:** 2024-06-10

**Authors:** Kaiqi Yang

**Affiliations:** School of Physical Education, Guizhou Normal University, Guiyang, China

**Keywords:** workload, game quarter, playing position, basketball, monitoring training load

## Abstract

**Purpose:**

This study aims to explore the variations in external and internal loads on a quarter-by-quarter basis among professional Chinese basketball players. It emphasizes the crucial impact of these variations on optimizing athletic performance and match strategies.

**Method:**

An observational longitudinal study design was employed, involving sixteen male players from the National Basketball League during the 2024 season in China. Data collection was facilitated through the use of Catapult S7 devices for measuring external loads and session ratings of perceived exertion (sRPE) for assessing internal loads. Linear mixed-effects models were utilized for the statistical analysis to identify differences in workload intensities across game quarters based on player positions. The Pearson correlation coefficient was used to examine the relationship between external and internal load throughout the game.

**Results:**

The analysis uncovered significant positional differences in workload intensities across game quarters. Guards were found to have a higher PlayerLoad™ (PL) per minute in the first quarter, while centers demonstrated an increase in high-intensity accelerations and jumps in the fourth quarter. Furthermore, a significant moderate correlation between sRPE and PL was observed across all game quarters, indicating a link between physical exertion and athletes’ perceptions of effort.

**Conclusion:**

The study offers new insights into the dynamic physical demands faced by basketball players and the importance of using both objective and subjective measures for a comprehensive assessment of athlete performance and wellbeing. The findings underscore the interconnectedness of physical exertion and athlete perception, providing a foundation for future research and practical applications in the field of basketball science.

## 1 Introduction

Monitoring training loads in basketball has emerged as a critical aspect of athletic performance optimization. This methodology distinguishes between external and internal loads, providing a comprehensive assessment of competition demands. External loads measure physical activities like distance covered and jumps, whereas internal loads assess the athlete’s physiological and psychological responses, such as heart rate and rating of perceived exertion (RPE) (Fox et al., 2017; West et al., 2021). This distinction facilitates the creation of customized training and recovery plans, aiming to improve performance and reduce injury risks, thereby underscoring the importance of understanding training loads in basketball (Conte et al., 2018).

Recent technological advancements have highlighted the importance of monitoring both external and internal loads during basketball competitions, offering insights into how these factors affect performance based on player position (Power et al., 2022). For example, García et al. (2020) observed that guards and forwards cover greater distances than centers, who show superior performance in peak velocity and jumps. In addition, centers also presented more moderate increased values in collisions (impacts >8G) than guards and forwards, which can complement the information presented above to describe the specific physical demands of the playing position (García et al., 2020). Similarly, the literature suggests that centers’ higher body mass presents challenges in acceleration, leading to a slower speed increase and a longer time to reach target velocities (Schelling and Torres-Ronda, 2013; Reina et al., 2020). Notably, the number of accelerations and decelerations per minute are related with high intensity neuromuscular efforts and showed a certainly moderate/large decrease for all playing positions in the last quarter (Vázquez-Guerrero et al., 2019). While basketball professionals can customize training programs according to the physical characteristics typical of player positions to enhance readiness for competition, it is crucial to acknowledge the dynamic nature of basketball. Consequently, understanding the physical variations experienced by players across different quarters of a game takes on greater significance.

The previous studies found external load variations over four game quarters, identifying significant (Vázquez-Guerrero et al., 2019; Reina et al., 2020; Alonso Pérez-Chao et al., 2022; Askow et al., 2023) differences in distance covered and PlayerLoad™ (PL) between the first and last quarters (García et al., 2020). In particular, the second quarter had the lowest PL values, whereas the fourth quarter had the lowest PL·min values (Askow et al., 2023). Similarly, Reina et al. (2020) and Vázquez-Guerrero et al. (2019) complemented these findings by demonstrating that forwards exhibited the greatest variability in external load, whereas guards maintained the most consistency throughout the game, being notably more active in the opening quarter and centers increasing their activity by the fourth quarter. However, these studies primarily involve youth and professional players from Europe and America (Vázquez-Guerrero et al., 2019; García et al., 2020; Reina et al., 2020; Alonso Pérez-Chao et al., 2022), leaving a gap in understanding these dynamics among players of other levels and nationalities, such as elite Chinese basketball players in the National Basketball League.

Understanding the relationship between external and internal loads provides valuable insights for coaches to design more effective training plans and game strategies. Scanlan et al. (2014) reported a significant moderate relationship between external training load and session Rating of Perceived Exertion (sRPE), as well as a strong correlation between external training load and the Summated Heart Rate Zone (SHRZ) model. Likewise, Svilar and Jukić (2018) identified a significant correlation between external load variables and sRPE, with sRPE exhibiting a strong association with various load metrics. In contrast, competition settings yielded mixed results. Fox et al. (2020) found a significant correlation between Player Load (PL) and both heart rate zones and sRPE during basketball competition, while other studies reported no significant correlation between external and internal load parameters (Willberg et al., 2022). Importantly, the relationship between external and internal loads across game quarters remains underexplored, underscoring the need for further investigation in this area.

This study aims to examine the changes in external and internal loads and their interrelation throughout basketball competitions among Chinese professional players. By addressing this research gap, we hope to provide valuable insights into the performance optimization strategies for elite Chinese basketball athletes. The expected outcomes will enrich global basketball science discussions and offer practical implications for improving training methodologies, athlete performance, and wellbeing. Anticipating significant load fluctuations across game quarters, this research lays the foundation for future explorations and practical applications in basketball science.

## 2 Materials and methods

### 2.1 Subjects

Sixteen professional, male basketball players (age: 27.2 ± 1.35 years; height: 198.4 ± 6.35 cm; body mass: 98.4 ± 12.7 kg) volunteered to participate in this study. Players belonged to the same team in the Men’s National Basketball League, which is a second-tier, state-level Chinese basketball competition. These players were routinely monitored at the request of the coaching staff, as it was anticipated these individuals would regularly receive playing time during games. Participants not receive substantial playing time were excluded from this investigation due to a lack of monitoring. Before the initiation of the study, all participants underwent a thorough health evaluation to confirm their eligibility, ensuring the absence of any injuries or medical conditions that could pose a risk to their safe participation. This process was followed by the acquisition of their voluntary, written informed consent. The research methodologies employed in this study received approval from the Ethics Committee of Guizhou Normal University according to the ethical guidelines of the Helsinki Declaration.

### 2.2 Design

This research employed an observational, longitudinal study design to assess both external and internal workloads throughout the competitive season’s in-season phase (2023/24 season). A 1-week pre-season period served as an acclimatization phase, allowing players to familiarize themselves with the monitoring protocols, though data from this period were excluded from the final analysis. Monitoring was conducted for all matches throughout the 12-week in-season phase. Over the course of the season, participants engaged in a total of 18 matches, scheduled between Saturday and Sunday on a weekly basis at both home and away venues, with the frequency of matches ranging from zero to three per week. Each match was structured into four-quarters, each lasting 10 min.

### 2.3 Procedures

Prior to each game, each player was assigned with a Catapult S7 device (Catapult Innovations, Melbourne, Australia) to ensure consistency in the data collection. The Catapult S7 device consisted of a 100 Hz tri-axial accelerometer, tri-axial gyroscope, and magnetometer and captured the multidirectional movements of the basketball players. The device was worn on the trunk between the shoulder blades at approximately the C7-T1 level in an anatomical harness (Olthof et al., 2021).

Throughout all matches, data from microsensors were meticulously recorded in real-time and subsequently transferred to a personal computer for detailed analysis via specialized software (OpenField, version 3.10.5; Catapult Innovations, Melbourne, Australia). In alignment with established methodologies, the analytical process omitted data pertaining to warm-up activities while incorporating periods of rest (for instance, breaks, timeouts, and substitutions within the competition) to accurately assess the comprehensive demands of each quarter (Fox et al., 2022).

To minimize disruption to the players and the game, internal load data were collected within 5 min of completing each quarter. Specifically, each player, separate from their peers, gave an individualized RPE to a member of the research team using Borg’s Category (CR-10) Ratio Scale (Borg et al., 1987).

Quantification of external workload was conducted using the accelerometer feature of the microsensors. The volume of external workload was gauged by PL, a unique metric formulated by sampling accelerometer data at 10 Hz. PL quantifies the cumulative workload as the square root of the aggregate of the squared changes in acceleration along the transverse (x), coronal (y), and sagittal (z) planes, amplified by a constant of 0.01. The reliability of PL, as evidenced by a coefficient of variation (CV) ranging between 0.9% and 1.9%, has been corroborated in the context of team sports (Barrett et al., 2014). In addition, external workload was assessed using various inertial movement analysis (IMA) variables derived from the inertial sensors (tria-xial accelerometer and tri-axial gyroscope) and identified based on the direction traveled by players. Specifically, accelerations (−45°–45°), decelerations (−135°–135°), changes-of-direction (COD; −135° to −45° for left and 45°–135° for right), and jumps (0–40 cm). Furthermore, the number of low-intensity events per minute, medium-intensity events per minute, and high-intensity events per minute was calculated.

For accelerations, decelerations, COD, and low-, medium-, and high-intensity events were defined as 1.5–2.5 m·s^−2^, 2.5–3.5 m·s^−2^, and >3.5 m·s^−2^, respectively. Jumps were determined via proprietary algorithms and classified as low-, medium-, and high-intensity events using jump height cutoffs of <20 cm, 20–40 cm, and >40 cm, respectively. The reliability (coefficient of variation [CV] = 3.1%–6.7%) of the IMA-derived external workload variables assessed has been previously supported in team sports (Luteberget et al., 2018). Furthermore, internal workload was evaluated subjectively using sRPE, which involved multiplying the individualized RPE by session duration (Foster et al., 2001).

### 2.4 Statistical analysis

Descriptive statistics are presented as mean ± SD. A linear mixed-effects model was used to model the main and interactive effects using R Studio (Version 4.2.3, R Core Team). “Quarter” (Q1, Q2, Q3 or Q4) and “position” (Center, Forward, or Guard) were treated as the fixed effects, whereas the random effects were “ID Player” and “match-code.” Differences were divided by the square root of the sum of the intercept and residual variance components in the model to determine a standardized effect size (ES) for each difference between categorical fixed factors. Effect size and confidence intervals (ES ± 90% CI) were calculated to quantify the magnitude of pairwise differences. Thresholds for effect sizes statistics were <0.20, trivial; 0.20–0.59, small; 0.6–1.19, moderate; 1.20–1.99, large; and >2.0, very large (Hopkins et al., 2009). In addition, the relationship between sRPE and PL was assessed with Pearson’s (r) and the following criteria used to interpret the magnitude of the correlation measures: <0.10, trivial; 0.10–0.29, small; 0.30–0.49, moderate; 0.50–0.69, large; 0.70–0.89, very large; and 0.90–1.00, nearly perfect (Hopkins et al., 2009). If the 90% CI overlapped positive and negative values, the magnitudes were considered unclear. Finally, A decision tree analysis was conducted using the Classification and Regression Tree (CART) algorithm. CART was chosen for its binary tree structure, where each node split is optimized based on a single variable. The model’s depth was dynamically determined, with no pre-set maximum, allowing the algorithm to adjust its complexity according to the inherent patterns present in the dataset. In contrast to the Chi-square Automatic Interaction Detector (CHAID) algorithm, which imposes constraints such as predefined minimum sizes for parent and child nodes and limits iterations to 100, our CART implementation featured no such restrictions. This afforded a more organic and natural development of the tree structure. Additionally, unlike CHAID analysis, our methodological framework did not rely on the “minimum change in expected cell frequencies” parameter to drive the splitting process. In our CART implementation, Gini impurity was selected as the criterion for splitting nodes. This metric quantifies the probability of an incorrectly classified element being picked at random, and it helped determine the most favorable bifurcation point at each node. This approach offered a nuanced perspective on the data, facilitating a comprehensive exploration of the variables that classify game shifts in basketball matches. For ease of interpretation, scalable visualizations of the resulting decision trees were created using the “pybaobabdt” library. These visualizations provided an intuitive understanding of the tree’s structure and the decision-making process behind each node split, greatly aiding in the comprehension and explanation of the model’s outcomes (Shi et al., 2024). The alpha level was set at *p* ≤ 0.05.

## 3 Results

The descriptive analysis of external and internal workload measures is presented in Table 1.

**TABLE 1:** Descriptive statistics of the external and internal load according to playing position across game quarter.

Variables	First quarter			Second quarter			Third quarter			Fourth quarter			
	Guards	Centers	Forwards	Guards	Centers	Forwards	Guards	Centers	Forwards	Guards		Centers	Forwards
sRPE	104 ± 62	77 ± 26	88 ± 37	102 ± 52	72 ± 35	113 ± 49	103 ± 55	72 ± 30	114 ± 56	110 ± 49		104 ± 63	114 ± 54
PlayerLoad	112 ± 53	99 ± 42	104 ± 47	115 ± 51	91 ± 43	96 ± 38	102 ± 42	84 ± 35	93 ± 34	116 ± 56		116 ± 63	115 ± 66
PlayerLoad·min	11.99 ± 2.09	10.08 ± 1.48	10.66 ± 2.18	11.17 ± 1.70	9.98 ± 1.64	10.41 ± 1.78	11.39 ± 1.88	10.49 ± 1.63	10.91 ± 2.03	10.64 ± 2.52		10.17 ± 2.61	9.84 ± 1.69
IMA Jump Low	4.4 ± 2.8	4.0 ± 2.7	5.7 ± 5.2	4.0 ± 2.9	5.1 ± 3.6	5.2 ± 4.7	4.8 ± 2.7	3.6 ± 2.7	6.0 ± 4.4	4.0 ± 2.9		6.1 ± 4.3	7.0 ± 7.8
IMA Jump Medium	2.61 ± 1.81	3.92 ± 2.02	4.37 ± 2.98	3.10 ± 2.51	5.33 ± 3.06	4.24 ± 2.82	3.13 ± 1.94	4.00 ± 2.49	4.07 ± 2.84	3.16 ± 1.62		5.50 ± 3.34	4.61 ± 4.21
IMA Jump High	2.68 ± 2.02	2.38 ± 2.02	1.96 ± 1.34	2.16 ± 2.10	2.83 ± 2.48	1.48 ± 1.30	2.47 ± 2.08	2.73 ± 2.17	2.00 ± 1.68	2.64 ± 2.33		4.50 ± 3.48	1.52 ± 0.73
High-intensity accelerations	3.07 ± 2.42	3.77 ± 2.68	2.30 ± 1.59	2.77 ± 2.43	4.58 ± 3.37	3.00 ± 2.52	2.03 ± 1.77	3.64 ± 1.91	2.93 ± 2.18	2.32 ± 1.93		6.10 ± 4.95	3.13 ± 2.69
High-intensity decelerations	3.29 ± 2.11	2.46 ± 1.81	2.85 ± 2.03	3.29 ± 2.75	2.75 ± 1.48	1.97 ± 1.59	3.03 ± 1.81	2.91 ± 1.30	2.46 ± 1.79	2.56 ± 2.00		3.20 ± 2.04	2.87 ± 2.40
IMA COD Left	3.43 ± 2.75	2.54 ± 1.45	3.15 ± 2.07	3.23 ± 2.17	3.42 ± 2.64	3.21 ± 2.27	3.17 ± 2.15	2.27 ± 2.15	2.00 ± 1.49	2.68 ± 2.25		3.50 ± 2.27	3.00 ± 2.83
IMA COD Right	4.89 ± 3.14	3.23 ± 2.35	3.85 ± 3.03	3.90 ± 2.77	3.08 ± 2.31	2.86 ± 2.36	3.00 ± 2.49	3.27 ± 2.10	3.11 ± 2.42	3.72 ± 2.85		2.90 ± 2.23	3.87 ± 3.20
Low-intensity IMA events·min	60 ± 13	61 ± 9	62 ± 22	57 ± 14	60 ± 12	57 ± 17	57 ± 11	66 ± 10	61 ± 16	54 ± 20		59 ± 16	52 ± 15
Medium-intensity IMA events·min	3.32 ± 1.02	3.57 ± 0.92	3.13 ± 1.61	2.93 ± 0.82	3.45 ± 1.06	2.93 ± 1.20	3.34 ± 0.96	3.26 ± 0.73	3.00 ± 1.12	2.95 ± 1.32		3.47 ± 1.36	2.65 ± 0.96
High-intensity IMA events·min	1.72 ± 0.56	1.50 ± 0.71	1.51 ± 0.68	1.40 ± 0.52	1.82 ± 0.62	1.35 ± 0.68	1.52 ± 0.57	1.84 ± 0.71	1.43 ± 0.68	1.22 ± 0.55		1.73 ± 0.50	1.29 ± 0.70

Abbreviations: sRPE, session rating of perceived exertion; COD, change of direction; IMA, inertial movement analysis.

**FIGURE 1:**
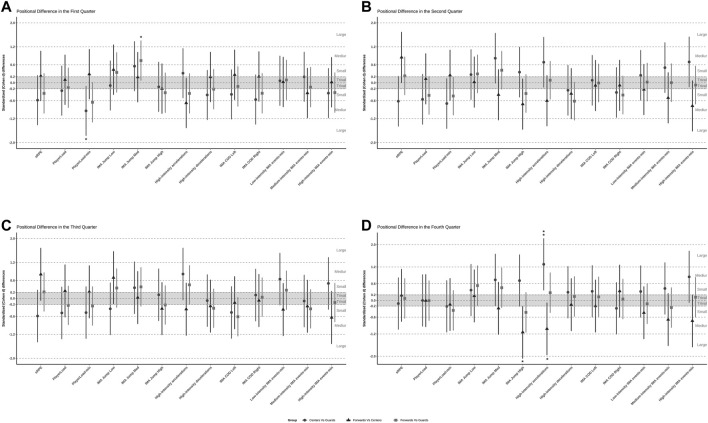
Standardized (Cohen’s d) differences computed variables according to playing position across game quarter. Note: p* <0.05; **p < 0.01; ***p < 0.001. **(A)** Positional differences in internal and external load in the first quarter. **(B)** Positional differences in internal and external load in the second quarter. **(C)** Positional differences in internal and external load in the third quarter. **(D)** Positional differences in internal and external load in the fourth quarter.

Figure 1 provides a detailed representation of the positional differences in both external and internal loads from the first quarter to the fourth quarter during basketball competition. In the first quarter, guards exhibited significantly higher Player Load (PL) per minute compared to centers (*p* < 0.05; ES = −0.94; Moderate). Conversely, guards displayed a significantly lower frequency of medium-intensity jumps than forwards (*p* < 0.05; ES = 0.74; Moderate), highlighting notable positional variations in performance metrics.

Interestingly, no significant differences in external and internal loads were observed between positions during the second and third quarters, suggesting a convergence in performance metrics across these periods. However, the fourth quarter presented a different trend, where centers showed a significantly higher frequency of high-intensity jumps than forwards (*p* < 0.01; ES = −1.14; Moderate). Furthermore, centers demonstrated a higher number of high-intensity accelerations compared to both guards (*p* < 0.01; ES = 1.30; Large) and forwards (*p* < 0.05; ES = −1.01; Moderate). These positional discrepancies in the fourth quarter underscore the varying physiological demands across player roles, especially in high-intensity activities.

**FIGURE 2:**
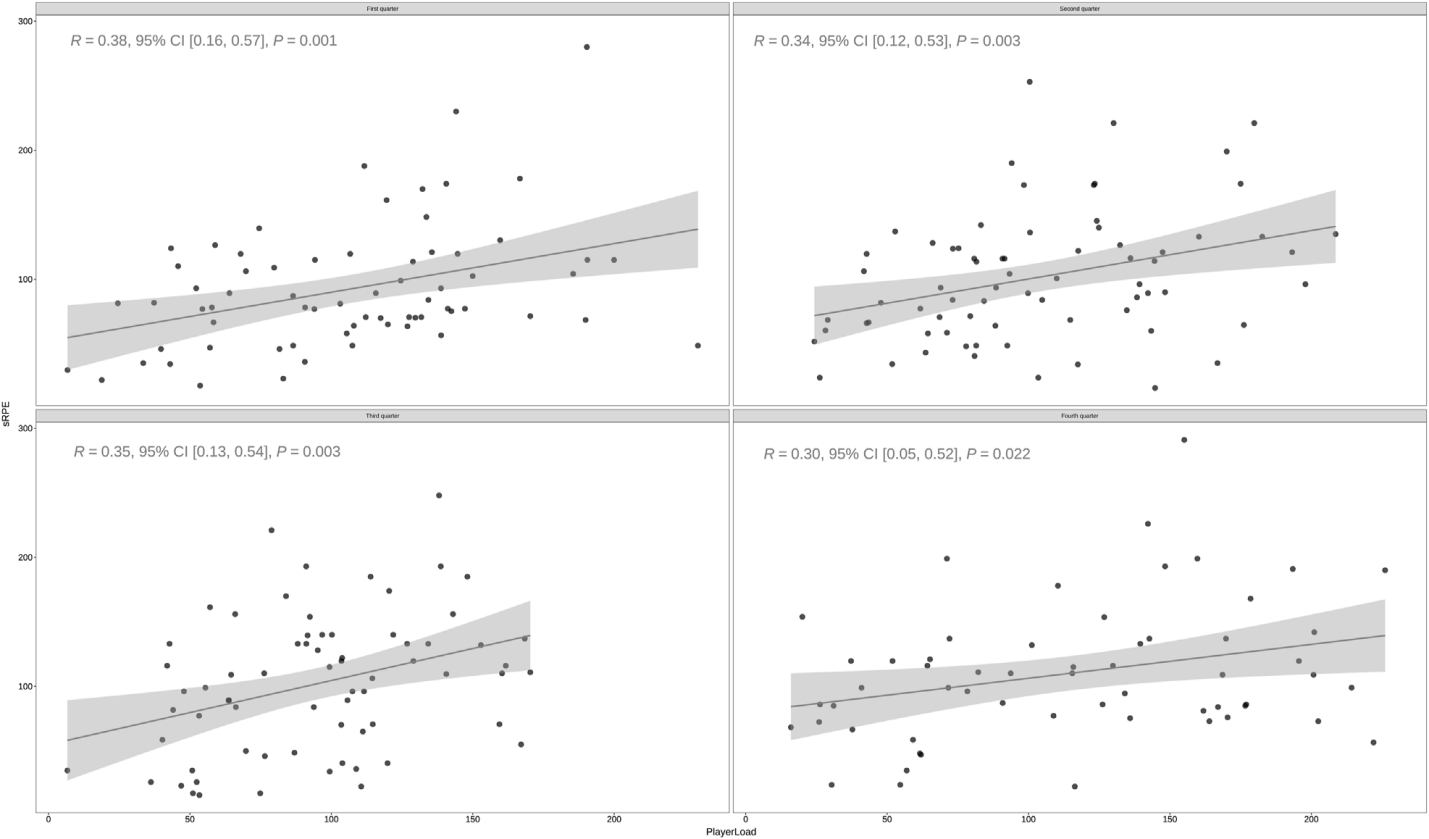
The correlations between PlayerLoad™ and sRPE across the four-quarter among Chinese basketball players. Note: The dashed bolded black line indicates the correlation for the overall model; sRPE, session rating of perceived exertion.

Figure 2 presents the correlation values with 95% confidence intervals (CIs) for all relationships between external and internal workload variables. Across all game quarters, significant correlations (*p* < 0.05) were found between external and internal workload variables during basketball competition. Notably, the relationship between session Rating of Perceived Exertion (sRPE) and Player Load (PL) was consistently moderate throughout the game quarters: first quarter (*p* < 0.01; r = 0.38; 95% CI [0.16, 0.57]; Moderate), second quarter (*p* < 0.01; r = 0.34; 95% CI [0.12, 0.53]; Moderate), third quarter (*p* < 0.01; r = 0.35; 95% CI [0.13, 0.54]; Moderate), and fourth quarter (*p* < 0.05; r = 0.30; 95% CI [0.05, 0.52]; Moderate). This consistent association reinforces the strong relationship between perceived exertion and objective external load metrics, emphasizing the value of sRPE as a reliable indicator of overall workload in competitive basketball settings.

**FIGURE 3:**
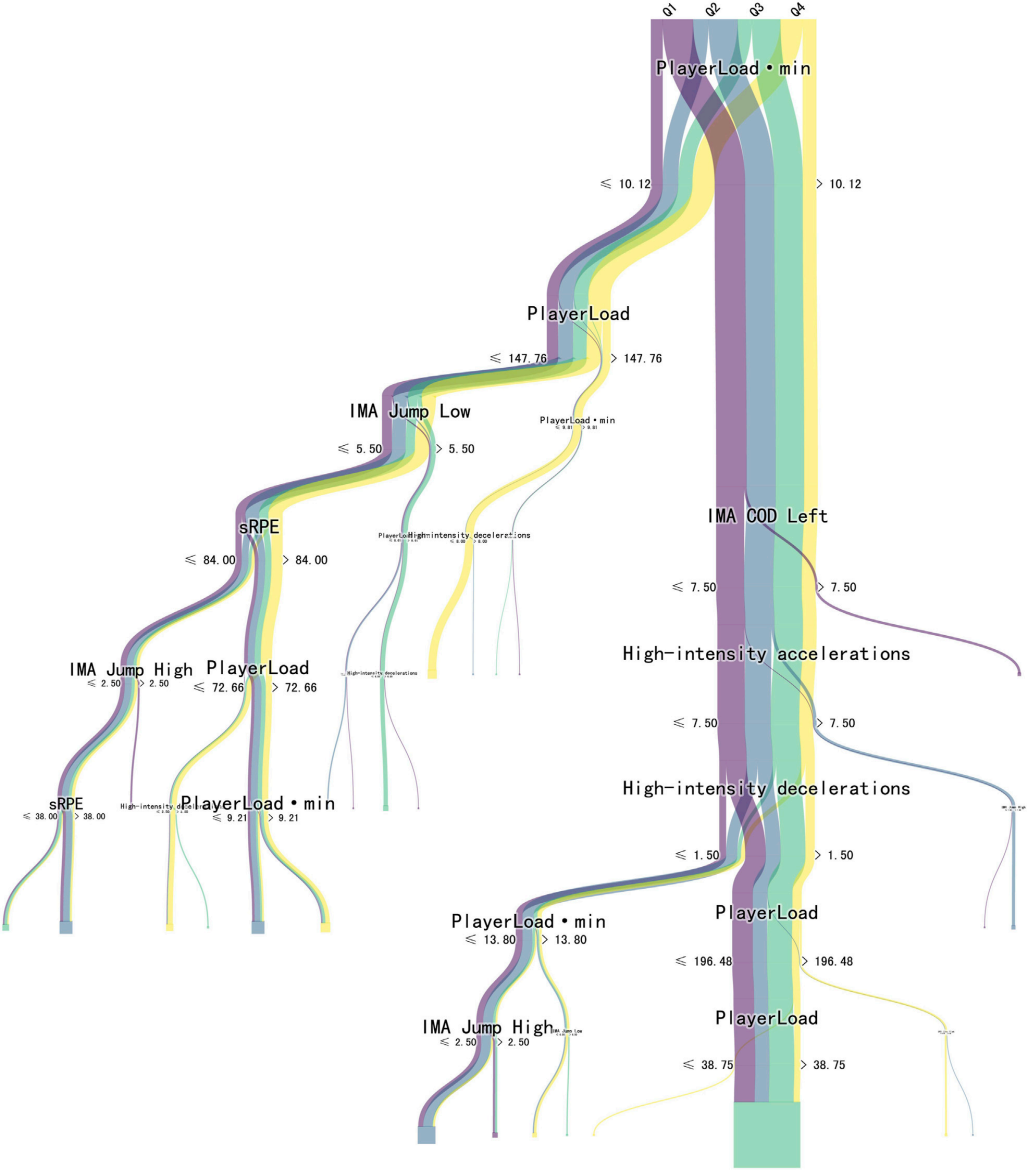
Visualization of Classification and regression tree from the first quarter to fourth quarter. Note: Q1, first quarter; Q2, second quarter; Q3, third quarter; Q4, fourth quarter.

The CART analysis based on the top six layer presented in Figure 3 identifies several pivotal nodes influencing the dependent variable, interpreted in the context of performance metrics across the four-quarters (Q1 to Q4) of a basketball game.

The PL per minute emerged as a significant node, branching at a threshold of 10.12. More critically, the CART algorithm delineates a substantial bifurcation for “PL” instances at a threshold greater than 147.76. The thickness of the corresponding yellow line in the figure accentuates a higher frequency of such elevated PL occurrences predominantly in the final quarter, Q4. Furthermore, sRPE also represents a critical node, dividing at a value less than or equal to 84.00. This bifurcation indicates a significant correlation between athletes’ perceived exertion and the outcome variable. It could reflect a shift in psychological and physiological stress that players experience, especially during high-intensity phases as the game advances. Similarly, high-intensity deceleration is also a decisive node, splitting at less than or equal to 1.50, suggesting that the frequency or intensity of such decelerations during gameplay significantly impacts the outcome variable. Each node is temporally connected, showcasing a dynamic interplay of these factors as the game unfolds. Notably, there is a discernible shift in how these factors interact and impact player performance in different game stages, with a marked emphasis on the latter stages.

## 4 Discussion

The aim of our study was to investigate the variations in external and internal workloads and their interrelationships across different quarters of basketball matches among professional basketball players in China. Our main finding indicated that guards experience a higher PlayerLoad™ (PL) per minute in the first quarter compared to centers, highlighting the intense initial activity level required of these positions. Conversely, in the final quarter, centers exhibit greater involvement in high-intensity accelerations and jumps than their teammates, underscoring the pivotal role of physicality in the game’s critical moments. Additionally, our study identifies periods of relative workload stability in the second and third quarters, suggesting a tactical balance achieved by players. The significant correlations between session ratings of perceived exertion (sRPE) and objective workload measures across all quarters further underscore the interconnectedness of physical exertion and athlete perception. This research not only advances our understanding of the sport’s physical demands but also provides a foundation for developing targeted training and recovery protocols that cater to the specific needs of each player position.

The higher PL per minute observed in guards compared to centers during the first quarter suggests high-intensity engagement in the game’s initial stages, likely attributed to their role in establishing game tempo and executing aggressive defensive strategies outside the three-point line (Heishman et al., 2019). Previous investigations have drawn similar conclusions, suggesting that entering the game in the first quarter is more physically demanding for guards, who often need to control the game pace and adapt to the competition environment (Vázquez-Guerrero et al., 2019). This search and attunement likely result in varied decision-making and movement patterns (Vázquez-Guerrero et al., 2019). Moreover, the decreased jump medium for guards compared to forwards might indicate a strategic preservation of energy for later stages of the game or a tactical focus on perimeter play, requiring less vertical movement but more agility and speed. This finding is supported by Reina et al. (2020), who found that guards presented a more stable performance throughout the game, whereas forwards showed the highest level of variability, possibly due to their required participation in all game phases (offense, defense, and transitions). Notably, although guards presented a higher PL per minute and lower vertical movement compared to centers and forwards, the importance of the first quarter for practitioners should not be overlooked. Combining the current evidence with the insights from Khoramipour et al. (2021), it’s evident that the initial phase of a basketball match is particularly demanding on players’ anaerobic energy systems. This is primarily due to the intense and frequent shuffling, movements, and ball-handling activities that occur mostly in the first quarter, leading to a significant increase in lactate production when compared to the latter half of the game. Such early-game external load serves as a catalyst for metabolic adaptations, emphasizing the critical energy requirements needed to meet the high-intensity demands of the opening quarter.

Our study found no difference in external and internal loads in the second and third quarters during match play. This result aligns with findings by Scanlan et al. (2015), found that suggesting that coaches and players often make tactical adjustments during these quarters based on the game’s flow and the opponent’s strategies observed in the first quarter. These adjustments, which might involve changing defensive setups (e.g., switching from man-to-man to zone defense) or altering offensive tactics (e.g., increasing the use of pick-and-roll plays), can lead to a more balanced exertion among players as teams seek to exploit weaknesses in their opponents’ setups or adjust to their strengths (Ben Abdelkrim et al., 2010; Bullock et al., 2018). From a physiological standpoint, players enter these quarters having already warmed up and acclimated to the game’s intensity. The initial surge of adrenaline and energy expenditure in the first quarter gives way to a more measured approach as players manage their energy reserves (Khoramipour et al., 2021). Coaches should note that the body’s energy systems, including the aerobic and anaerobic pathways, are utilized more efficiently as the game progresses, allowing players to maintain a consistent performance level. Thus, game load management is crucial for maintaining performance levels throughout the game, leading to a stabilization of both external (e.g., distance covered, sprints) and internal (e.g., heart rate, perceived exertion) workload indicators (Conte et al., 2018; Fox et al., 2021; Kamarauskas and Conte, 2022). Most importantly, coaches play a pivotal role in making real-time decisions that balance the need for immediate competitiveness with the strategic conservation of energy for the later stages of the game. For example, utilizing their benches to give starters intermittent rest periods helps teams keep the game’s intensity high without overburdening their key players, ensuring they have the energy reserves needed for the final push in the fourth quarter (Gomez et al., 2016). Therefore, the stability in external and internal loads during the middle quarters reflects a multifaceted approach to managing the game, encompassing tactical adjustments, physiological considerations, strategic balance, and adaptation. These factors collectively contribute to sustaining high performance levels and are essential components of successful basketball strategies.

This trend shifts noticeably in the final quarter, where centers exhibit increased engagement in high-intensity activities, including jumps and accelerations, surpassing both guards and forwards, highlighting the pivotal role of physical presence and power in the game’s critical moments. In the concluding stages of basketball matches, centers assume a critical role due to strategic shifts towards gameplay that emphasize scoring from positions close to the basket and maximizing rebounds (Vázquez-Guerrero et al., 2019). This result is supported by Reina et al. (2020), suggesting that centers engage more frequently in high-intensity actions such as jumping and accelerating, utilizing their physical attributes and energy reserved from the initial stages of the game. Their augmented participation is a deliberate strategic choice and a physiological tactic, as their physical preparation enables them to exert significant force during the latter part of the game. Psychologically, the involvement of centers in crucial moments serves to deter the opposition and elevate their own team’s confidence through decisive plays. Experienced centers leverage their knowledge for leadership, directing the team with strategic offensive decisions and stabilizing the defense (Ben Abdelkrim et al., 2010; Manuel Clemente et al., 2019; Vázquez-Guerrero et al., 2019). This comprehensive approach highlights the importance of physical strength and presence in determining the outcome of the game’s crucial moments, underlining the central players’ role in securing an advantage for their team in the final quarter.

Our results reveal a significant correlation between sRPE and PL across all game quarters, indicating a moderate relationship between these internal and external workload indicators in basketball. This result is linked with Espasa Labrador et al. (2021), suggesting that players’ perceived exertion levels moderately align with the objective measures of physical workload (PL) throughout the game, offering a refined perspective on the workload experienced by players. Furthermore, Fox et al. (2020) provided complementary insights by examining the relationships between various workload indicators during basketball training and games in a semi-professional male context. Their research highlighted that PL was more closely associated with internal workload indicators, especially summated heart-rate zones (SHRZ) and sRPE, compared to other external workload indicators, indicating that PL effectively predicts the physiological and psychological stress experienced by players (Fox et al., 2020). Our study adds to this understanding by demonstrating how perceived exertion (an internal load indicator) consistently correlates with an objective external load metric (PL) throughout a game. This consistency suggests that perceived exertion is a reliable indicator of actual workload, adding a crucial dimension to optimizing basketball performance. Moreover, Fox et al. (2020) also discovered stronger correlations during training than in games, pointing to possible differences in workload perception and response across contexts. Taken together, our study underscores the importance of integrating both objective (PL) and subjective (sRPE) workload measures to fully grasp their impact on players. This approach is vital for coaches and sports scientists aiming to customize training and recovery programs to meet individual player needs and experiences.

The CART analysis particularly emphasized the temporal progression and the increased load in the latter stages of a basketball game. The emergence of PL per minute as a pivotal node suggests that the distribution of exertion over the game duration is not uniform. The decision point at a threshold greater than 147.76 for PL instances indicates a critical juncture at which player performance may either peak or diminish, possibly due to fatigue. The conspicuous thickness of the yellow line in the CART diagram for the final quarter (Q4) underscores the augmented load players face as the game nears conclusion. This finding is in tandem with the notion that the final quarter’s heightened physical demands could exacerbate the risk of performance decrement or injury (Vázquez-Guerrero et al., 2019; Askow et al., 2023). The sRPE as a branching node speaks to the psychological and physiological stress factors, which are integral to understanding player condition and readiness. The split at ≤84.00 can be interpreted as a threshold where players’ perceived exertion begins to have a tangible impact on the measured outcome. This is particularly relevant for coaching strategies that aim to manage player workload and optimize performance through regulated recovery periods (Zhang et al., 2019). Similarly, the node representing high-intensity deceleration further adds to the discussion on the physical demands placed upon players during a game. The physiological cost of high-intensity activities, including rapid decelerations, could contribute to accumulative fatigue (Hewit et al., 2011), which is evidenced by its significant impact on the quarter change at the ≤1.50 split. Such insights are crucial for the development of training and conditioning programs that aim to bolster players’ resilience to high-intensity activities throughout the game (Reina et al., 2019; McBurnie et al., 2022). The decision tree analysis provides insights into how different levels of “PlayerLoad” affect specific game movements and overall player performance. This information is vital for basketball players, as it aids them in comprehending player patterns, refining game strategies, and ultimately elevating their on-court performance.

Notwithstanding the valuable insights garnered from our study, it is imperative to acknowledge its limitations. One of the primary constraints relates to the sample size and demographic focus, which may limit the generalizability of our findings to wider populations and competitive levels. Additionally, while our analysis provides a robust examination of workload dynamics, it does not directly address the psychological or tactical dimensions of basketball performance, representing critical avenues for future investigation. Expanding the scope of research to encompass these areas, as well as incorporating more diverse and comprehensive datasets, will be essential for developing a more holistic understanding of game demands during game-play.

## 5 Conclusion

Our study contributes significantly to the field of basketball science by elucidating the quarter-by-quarter variations in external and internal loads among elite Chinese basketball players. The distinct workload profiles identified for different player positions across game quarters underscore the complex interplay between the physical demands of basketball and the athletes’ physiological and psychological responses. The findings highlight the need for tailored training and recovery protocols that account for these variations, thereby enhancing performance and reducing the risk of injury. Furthermore, the study’s emphasis on both objective (PL) and subjective (sRPE) measures of workload presents a holistic approach to athlete monitoring, advocating for the integration of these metrics in the development of optimized training regimens. PL per minute, sRPE, overall PL, and high-intensity deceleration are the primary factors determining the dynamic shifts in game quarters throughout the match. Limitations regarding the study’s sample size and demographic focus suggest avenues for future research, including investigations into the psychological and tactical dimensions of performance. By advancing our understanding of workload dynamics in basketball, this research offers valuable implications for coaches, athletes, and sports scientists aiming to elevate the standards of athletic preparation and performance in basketball.

## Data Availability

The raw data supporting the conclusion of this article will be made available by the authors, without undue reservation.
